# Leukocyte- and platelet-rich fibrin in cranial surgery: study protocol for a prospective, parallel-group, single-blinded randomized controlled non-inferiority trial {1}

**DOI:** 10.1186/s13063-023-07252-w

**Published:** 2023-03-23

**Authors:** Birgit Coucke, Anaïs Van Hoylandt, Johannes van Loon, Frank Van Calenbergh, Laura Van Gerven, Tom Theys

**Affiliations:** 1 Research Group Experimental Neurosurgery and Neuroanatomy and the Leuven Brain Institute, Department of Neurosciences, KU Leuven, Leuven, Belgium; 2 Allergy and Clinical Immunology Research Group, Department of Microbiology, Immunology & Transplantation, KU Leuven, Leuven, Belgium; 3 Department of Neurosurgery, University Hospitals Leuven, Leuven, Belgium; 4 Department of Otorhinolaryngology-Head and Neck Surgery, UZ Leuven, Leuven, Belgium; 5 Laboratory of Experimental Otorhinolaryngology, Department of Neurosciences, KU Leuven, Leuven, Belgium

**Keywords:** CSF leakage, Dura, Sealing, Prevention

## Abstract

**Background:**

CSF leakage is a major complication after cranial surgery, thus, adequate dural closure must be performed. Commercially available fibrin sealants are currently considered the gold standard for dural closure, but problems have been reported regarding safety, efficacy, and costs. This trial aims to investigate autologous leukocyte- and platelet-rich fibrin (L-PRF) as an alternative to commercially available fibrin sealants.

**Methods/design:**

This single-blinded, prospective randomized controlled interventional trial aims to demonstrate the non-inferiority of L-PRF compared to commercially available fibrin sealants for dural closure. This trial will include patients undergoing cranial neurosurgery (supratentorial and infratentorial) with intentional opening of the dura. Patients are randomized in a 1:1 fashion comparing L-PRF to commercially available fibrin sealants. The primary endpoint is postoperative CSF leakage within 12 weeks after surgery. Secondary endpoints are complications such as bleeding or wound infections. Additionally, a cost-effectiveness analysis is performed.

**Discussion:**

With this trial, we will evaluate the safety and efficiency of L-PRF compared to commercially available fibrin sealants.

**Trial registration:**

ClinicalTrials.gov NCT03812120. Registered on 22 January 2019.

**Supplementary Information:**

The online version contains supplementary material available at 10.1186/s13063-023-07252-w.

## Administrative information

We used the Standard Protocol Items: Recommendations for Interventional Trials (SPIRIT 2013) checklist when writing our report [1]. The numbers in curly brackets in this protocol correspond to the SPIRIT checklist item numbers.

**Table:** 

Title {1}	Leukocyte- and platelet-rich fibrin in cranial surgery: a prospective, randomized controlled trial
Trial registration {2a}	Clinicaltrials.gov (ID: NCT03812120) KU/UZ Leuven: S61460
Protocol version {3}	V8 dd 9–12-2021
Funding {4}	FWO TBM grant T003018N
Author details {5a}	Experimental Neurosurgery and Neuroanatomy, Department of Neurosciences, KU Leuven, Leuven, Belgium Department of Neurosurgery, UZ Leuven, Leuven, Belgium
Name and contact information for the trial sponsor {5b}	UZ Leuven Herestraat 49, 3000 Leuven, Belgium + 32 16 33 22 11
Role of sponsor {5c}	The sponsor has no role in the design of the study, study conduct, collection of the data, analysis, or writing of trial manuscripts
Date and version identifier {3} Date	Version
7–11-2018	Original (v1)
19–9-2019	Amendment no.1: Sample size correction, added detail to inclusion and exclusion criteria (v4)
5–2-2020	Amendment no.2: Addition of ICF translated in French and English (v4)
15–12-2020	Amendment no.3: Addition of quality control measures (v6)
24–11-2021	Amendment no.4: Specification of quality control measures (v8)

## Background {6}

A common complication after cranial surgery is cerebrospinal fluid (CSF) leakage. Our recently published systematic review, summarizing data from 113 studies, showed an average postoperative CSF leakage rate of 2.9% in supratentorial procedures, 5.8% in infratentorial procedures, and 4.1% in endoscopic transsphenoidal surgeries. Postoperative leaks may require revision surgeries and/or external CSF drainage [2]. Adequate dural closure is necessary to prevent CSF-related complications such as infection and intracranial hypotension. Currently, commercially available fibrin sealants are considered the gold standard for dural closure, due to their hemostatic, adhesive, and sealant properties. However, problems regarding efficacy, cost, and safety have been reported.

**Fig. 1:**
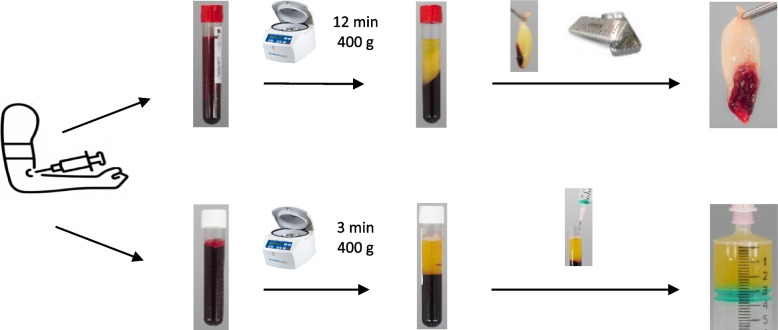
Leukocyte- and platelet-rich fibrin (L-PRF) preparation protocol. Top: membrane preparation after blood collection in glass tubes and centrifugation for 12 min (400 g), compression of the fibrin clot. Bottom: liquid L-PRF glue preparation after blood collection in plastic tubes and centrifugation for 3 min (400 g), collection of the upper layer in a sterile syringe

A possible alternative to commercially available fibrin sealants is autologous fibrin rich in platelets and leukocytes. The technique to produce such fibrin membranes was first described by Choukroun et al. in 2001 [3]. Leukocyte- and platelet-rich fibrin (L-PRF) is currently applied as an autologous biomaterial in various specialties, including oral and maxillofacial surgery and sports medicine [4]. The main advantage of L-PRF is that it is completely autologous, thereby minimizing the risk of immunological reactions. L-PRF is readily available as it is derived from the patient’s own blood, and accordingly, the method for preparation is non-invasive. Finally, L-PRF can be prepared “bedside” during surgical procedures by the operating room staff, within a 20-min time frame. For L-PRF membrane preparation, blood is collected in glass-coated tubes and centrifuged at 400* g* for 12 min (Fig. 1). During centrifugation, platelets are activated and the coagulation cascade is initiated. This results in a fibrin clot, which can be compressed into a membrane, for use as an onlay graft or cylinder to plug defects. These flaps can be placed on a defect, where the leukocytes and platelets that are incorporated in the fibrin network deliver growth factors to the surrounding tissue. Analogously, liquid L-PRF glue can be prepared in plastic-coated tubes. Because of the hydrophobic surface and shorter centrifugation time (3 min), the product maintains its native fibrinogen and only coagulates in contact with other tissues [5].

Application of L-PRF would improve wound healing and tissue regeneration, with a reduced infection risk. Apart from the uncomplicated preparation process, the low cost is an additional benefit of L-PRF. The cost of 8 blood tubes is approximately €12, which is very low compared to the price of commercially available fibrin sealants (€100–250) [6].

Given the myriad of theoretical advantages, the use of L-PRF is currently studied in numerous medical disciplines. In neurosurgery, it could play a role in the watertight closure of the dura. The application of L-PRF, in particular, in skull base reconstruction after endoscopic endonasal approaches, has already been described in a few retrospective research articles [7–9]. No major contra-indications were reported for L-PRF in endoscopic endonasal skull base reconstruction or craniectomies. Although L-PRF was reported as a promising biological, studies thus far concluded that larger, prospective trials are warranted to explore the possible applications of L-PRF. This clinical study aims to provide evidence in support of L-PRF in dural closure.

## Objectives {7}

The general aim of this study is to evaluate the potential of L-PRF in dural closure. More specifically, we are investigating L-PRF as an onlay graft on sutured dura in supratentorial and infratentorial surgeries. We aim to show the non-inferiority of L-PRF compared to the current standard practice. In addition, we want to demonstrate that L-PRF is more cost-effective than standard commercially available fibrin sealants. Risk factors and possible complications will be evaluated to validate safety.

## Trial design {8}

This study is a single-blinded, prospective randomized controlled trial with subjects randomized 1:1 in two parallel groups, i.e., experimental (L-PRF treatment) and active control (commercially available fibrin sealants).

In this study, the general objective is to show the non-inferiority of L-PRF as a closure technique compared to commercially available fibrin sealants in supra- and infratentorial surgery. This prospective randomized controlled trial was approved by the local Ethical Committee (UZ Leuven, S61460) and is registered at ClinicalTrials.gov (ID: NCT03812120) [2]. Recruitment started on 06 January 2020.

Demographic data, medical history, and surgical indications are reported prior to surgery. During the hospitalization (from 1 day preoperatively until the day of discharge), vital parameters such as body temperature and blood pressure are monitored, as well as medication intake. Patient’s quality of life (QoL) is questioned using the Research and Development 36-item Short Form Survey (RAND SF-36) and EuroQol Five Dimensions (EQ-5D) questionnaires.

**Fig. 2:**
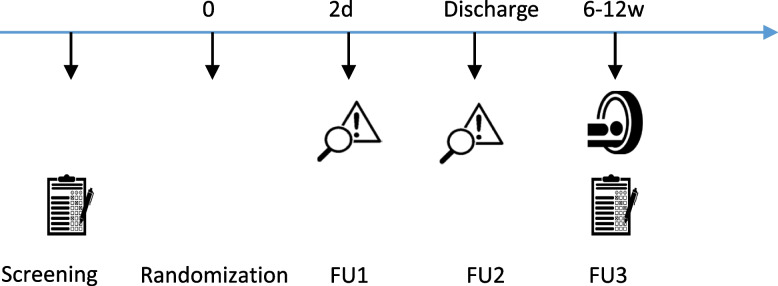
Flowchart of “L-PRF in cranial surgery, a prospective randomized controlled trial.” After obtaining informed consent, patients are screened and asked to complete quality of life (QoL) questionnaires (EQ-5D and RAND SF-36). During surgery (i.e., before dural closure), study subjects are randomized into the experimental arm (L-PRF) and the control arm (commercially available fibrin sealants). During hospitalization, patients are followed up with clinical controls after 2 days (FU1) and on the day of discharge (FU2). One outpatient follow-up is done between 6 and 12 weeks postop (FU3), with MRI imaging and completion of the QoL questionnaires

To perform a cost-effectiveness evaluation, all costs and effects will be collected. Additionally, patient’s general quality of life is assessed using the EQ-5D-3L Questionnaire prior to surgery and at the 6–12 weeks follow-up visit (FU3). Complications and adverse events are assessed at the time of surgery, 2 days postop (FU1), at hospital discharge (FU2), and at follow-up visits (FU3) (Fig. 2).

## Methods: participants, interventions, and outcomes

### Study setting {9} and participants {10}

This study is recruiting patients admitted to the Department of Neurosurgery of the University Hospitals Leuven (UZ Leuven) for elective neurosurgical procedures. Before initiation of any study procedure, participants (or their legal representative) must provide written informed consent (Additional file 2: Appendix 1). Inclusion criteria are patients undergoing cranial surgery with intentional opening of the dura (supratentorial and infratentorial approach) with a minimum age of 18 years (Table 1). Surgical indications include tumor resection and vascular (aneurysm, arteriovenous malformations) or functional (epilepsy surgery, microvascular decompression) neurosurgery. Pregnancy and participation in other clinical trials with study drugs or devices are exclusion criteria. Patients undergoing transsphenoidal surgery for sellar or parasellar lesions are not included, as we are currently performing a similar study on this subject, with more appropriate clinical follow-up for that type of lesion.

**Table 1:** Eligibility criteria

Inclusion criteria	Exclusion criteria
Age ˃ 18 years	Age ˂ 18 years
Supratentorial or infratentorial surgery	Transsphenoidal surgery
Informed consent signed	Pregnancy
	Participation in other clinical studies with drugs or medical devices

### Who will take informed consent? {26a}

Patients will be informed about the study by the neurosurgeon at the moment the elective surgery is planned. The information brochure is sent in advance by the study coordinator. Informed consent is obtained by trained study staff prior to surgery, i.e., on the day of hospital admission, usually 1 day before surgery. Specialized forms for interpreters are available, as well as specialized informed consent forms for patients not capable to give consent (legally authorized representative). In such cases, an individual’s capacity to consent is determined by the physician.

### Additional consent provisions for collection and use of participant data and biological specimens {26b}

There are no plans for additional studies using the data collected in this trial. No biological specimens are collected.

## Interventions

### Explanation for the choice of comparators {6b}

At present, commercially available fibrin sealants are considered the gold standard for dural closure. At UZ Leuven, Tisseel® (Baxter, Deerfield, IL, USA) and TachoSil® (Corza, Düsseldorf, Germany) are used as the standard of care for dural closure. Tachosil® is approved by the European Medicines Agency (EMA) and Food and Drug Administration (FDA) for this indication.

### Intervention description {11a}

The surgery is performed by a team of trained neurosurgeons according to local standards. The dura is primarily closed with sutures, and additional grafts can be used to close the remaining defects. For patients allocated to treatment with L-PRF, no commercially available fibrin sealants may be used for dural closure, but they can be used for hemostasis or to fixate Teflon® or vessels during microvascular decompression. Additional autologous (pericranium, muscle, fat grafts), allogenic (fascia lata), or synthetic (NeoDura®, Medprin Biotech GmbH, Frankfurt am Main, Germany) materials and hemostatic materials (cellulose sponges such as Spongostan® (Ethicon Biosurgery, Johnson and Johnson, New Brunswick, NJ, USA), Floseal® (Baxter, Deerfield, IL, USA), or Surgicel® (Ethicon Biosurgery, Johnson and Johnson, New Brunswick, NJ, USA)) can be used in both groups if deemed necessary by the surgeon.

L-PRF and liquid L-PRF glue are prepared during surgery. In all study subjects, L-PRF preparation is performed by the study coordinator to ensure protocol adherence. In this procedure, timing is particularly important to guarantee optimal membrane formation. Therefore, the arterial line is flushed, and immediately after, 20 mL of arterial blood is collected in a sterile syringe and transferred to two sterile 10-mL glass tubes (A-PRF®, Process for PRF, Nice, France), and instantaneously put in a centrifuge at 2700 rpm (400* g*) (IntraSpin®, Intra-Lock, Boca Raton, FL, USA). At the time of transfer to the glass tubes and start of centrifugation, a second 20-mL syringe is filled with arterial blood. The centrifugation process is interrupted as the second batch of collected blood is transferred to 10-mL glass tubes, to ensure precise timing to add the tubes into the centrifuge when decelerated. The centrifuge is instantly restarted at 2700 rpm for 12 min. If deemed necessary, one more 20-mL syringe can be collected to fill two more 10-mL glass tubes and added to the centrifuge in a similar fashion. After the centrifugation process, the tubes are inspected for the presence of a dense clot right above the red blood cell layer. If these are not present, the tubes are set aside for 10 min and inspected again. The tubes are opened and presented to the scrub nurse to remove the clot with sterile tweezers. The clot is placed in an Xpression Box® (Intra-Lock, Boca Raton, FL, USA) and gently compressed by placing the compression plate and the lid on top. After 5 min, the membranes are ready for surgical application by placing them on sutured dura. If necessary, the membranes can be left in the Xpression box® for up to 3 h until application.

Liquid L-PRF glue is prepared in a similar fashion. The arterial line is flushed, and 20 mL of blood is collected in a sterile syringe. The blood is then transferred to plastic tubes (S-PRF®, Process for PRF, Nice, France) and placed in the centrifuge for 3 min (2700 rpm/400* g*). If necessary, an additional 20 mL can be collected for liquid L-PRF glue preparation. After centrifugation, the tubes are opened and presented to the scrub nurse. The yellow upper layer is aspirated into a sterile syringe using a large-gauged transparent catheter-over-needle. Liquid L-PRF glue can be directly applied on L-PRF membranes placed on the dura. Alternatively, cellulose sponges or bone fragments can be immersed in the liquid L-PRF glue before placing them on the defect.

### Criteria for discontinuing or modifying interventions {11b}

After allocation to the experimental or control arm, the patient can still be excluded from the study in certain cases, specified as the perioperative exclusion criteria. A patient allocated to the control arm may be excluded if there is a clear indication for hypersensitivity reactions to commercially available fibrin sealants. Patients allocated to L-PRF may be excluded if the L-PRF preparation process fails or if the product is considered of insufficient quality. Subjects with other intraoperative findings identified by the surgeon that may preclude the conduct of the study procedure can be excluded as well, to ensure optimal patient care in the situation. For example, when the dura is exceptionally damaged, the surgeon may opt for multilayer reconstruction using multiple grafts, commercially available sealants, and autologous materials. Study participants can withdraw from the study at any moment, which implies all subsequent study procedures and data collection are discontinued.

### Strategies to improve adherence to interventions {11c}

The study coordinator is present during the intervention to ensure adherence to the allocation. As the intervention is administered at a single time point during surgery, no further strategies for intervention adherence are necessary.

### Relevant concomitant care permitted or prohibited during the trial {11d}

Patients with radiotherapy or chemotherapy scheduled within 7 days after the surgery are excluded. All other forms of treatment are permitted; however, patients are not allowed to participate in other clinical studies with investigational drugs or devices.

### Provisions for post-trial care {30}

In accordance with the Belgian Law relating to experiments on human persons (May 7, 2004), the sponsor shall assume, even without fault, the responsibility for any damages incurred by a study patient and linked directly or indirectly to the participation to the study and shall provide compensation therefore through its insurance.

## Outcomes {12}

The primary endpoint is the success rate of both techniques, which means the absence of a clinically relevant CSF leak at 12 weeks after surgery, i.e., any leak that needs surgical revision or any other intervention, e.g., puncture or aspiration, longer hospitalization, or repeat imaging. The success rate in both groups will be reported in proportions with a 95% confidence interval.

Secondary endpoints include an analysis of peri- and postoperative complications (CSF leakage, both clinically relevant (incisional leakage) and not clinically relevant (swelling, pseudomeningocele), surgical site infection and treatment-site bleeding in particular), as well as a cost-effectiveness evaluation. In addition, the study will compare the efficiency of L-PRF in supratentorial and infratentorial surgery, based on surgical approach and technique.

Peri- and postoperative complications will be obtained from the hospital databases. Each complication requiring additional treatment (surgical revision, intervention, or drug therapy) or investigation (clinical or imaging) or which requires or prolongs hospitalization will be considered. RAND SF-36 questionnaires will be filled out preoperatively (baseline) and 6–12 weeks post-surgery to evaluate patient-reported health concepts such as physical functioning, bodily pain, role limitations due to physical health problems, role limitations due to personal or emotional problems, emotional well-being, social functioning, energy/fatigue, and general health perceptions. The questionnaire is scored as indicated on the official website of RAND, adapted from the scoring described by Ware and Sherbourne [10]. The difference in the change in score to baseline will be compared between the groups, i.e., treated with commercially available fibrin sealants or treated with L-PRF.

For the cost-effectiveness analysis, the “healthcare payer” perspective will be adopted, which means that all direct treatment-related costs as well as costs related to follow-up will be included in the analysis. For the cost analysis, the following outcomes will be considered: used materials during surgery (units), surgery duration (minutes), intensive care hospitalization time (days/hours), duration of hospitalization (days), associated facility, and staff resources. These data will mainly be retrieved from the hospital records and requested at the hospital’s billing office, if necessary. The most recent unit prices at the time of analysis will be applied. Depending on the primary outcome, i.e., the effectiveness of dural closure, and the clinical effects of both treatments, analysis will be performed as cost-utility or as cost minimization [11, 12]. In addition, the use of EQ-5D-3L to calculate QALYs at 6–12 weeks postoperative will be explored to compare EQ-5D utility scores to other outcomes described above.

The outcomes will be assessed at five study visits within a timeframe of twelve postoperative weeks (Table 2), and a comparison will be made between the two groups considering the change to baseline. Data will be presented as mean and standard deviation or median and interquartile range for continuous variables (including body temperature, °C; blood pressure, mmHg; diameter of craniotomy, mm) and as proportions with 95% confidence intervals for categorical variables (complications, surgical indication, surgical approach). Baseline medical characteristics will be compared between the allocation groups in a similar fashion.

**Table 2:** Outcome assessment

Procedure	Visit 1: *screening*	Visit 2: *allocation*	Visit 3: *FU1*^a^	Visit 4: *FU 2*^b^	Visit 5: *FU 3*^c^
**Enrollment**					
Eligibility screen	X				
Informed consent	X				
Randomization		X			
**Interventions**					
Supratentorial/fossa posterior surgery		X			
Dura closure		X			
**Assessments**					
Medical history	X				
EQ-5D	X				X
RAND SF-36	X				X
MR imaging	X				X
Cost/material registration		X			
CSF leakage assessment			X	X	X
Wound healing assessment			X	X	X
Infection assessment			X	X	X
Adverse events		X	X	X	X

*FU* follow up

^a^Two days postoperative

^b^At hospital discharge

^c^Six to twelve weeks postoperative

### Participant timeline {13}

#### Screening

On the day of hospital admission (usually one day before surgery), informed consent is obtained, and the eligibility criteria are checked. Relevant medical history, medication use, vital parameters (blood pressure, temperature), and smoking and alcohol use are recorded. EQ-5D and RAND SF-36 questionnaires are completed.

#### Randomization

During surgery, the patients are randomized into the experimental (L-PRF) and control (commercially available fibrin sealant) groups.

#### Hospitalization

During their hospital stay, patients are evaluated 2 days after surgery (FU1) and on the day of discharge (FU2). The surgical site is assessed for signs of CSF leakage and/or infection. Medication administration and vital parameters are recorded.

#### Outpatient follow-up

At 6–12 weeks (FU3), the patient is seen at the outpatient department for another examination of the surgical site. CSF leakage, infection, and wound healing are assessed. Medication use is recorded, and EQ-5D and RAND SF-36 questionnaires are completed. Postoperative imaging is performed.

### Sample size {14}

Sample size calculation was performed according to the primary outcome, using an online power calculator for binary outcome non-inferiority trials (Sealed Envelope Ltd. available from: www.sealedenvelope.com). This is a non-inferiority trial with a binary outcome using a one-sided alpha of 0.05 and 80% power. The assumed success rate is 93.7% in the control arm, based on center-specific experience as well as the literature [13]. Data on the use of L-PRF for this indication is limited, but we assume a 95% success rate based on a prior feasibility study [9].

The statistical hypothesis for testing the treatment difference is presented as follows: H_0_: Δ ≤  − 0.05 tested against the alternative hypothesis and H_A_: Δ >  − 0.05, where Δ is the difference between the success rates of experimental and control condition and − 0.05 is the non-inferiority difference. The non-inferiority limit was set at 0.05 considering (1) reported CSF leakage rates without commercially available fibrin sealant averaging 17.2% [14] and (2) important additional benefits of L-PRF compared to commercially available fibrin sealants including the completely autologous nature (eliminating immune reactions), presence of immunologic cells and reduced costs.

Based on this power calculation, 334 patients need to be included, 167 in each group. A drop-out rate of 2–5% is expected due to loss of follow-up and non-adherence to the allocation or failure of L-PRF preparation. Therefore, 350 patients will be enrolled, 175 in each group.

### Recruitment {15}

In 2017, 400 patients underwent cranial surgery at UZ Leuven, and around 350 would have been potential candidates for this study. Considering the estimated enrollment rate of one out of three patients, we aimed to finish recruitment within 3 years. Due to the impact of the COVID-19 crisis, recruitment will be extended for one additional year. Patients will be recruited via the surgical tool of the electronic hospital record system. The planning tool is scanned weekly by the study coordinator and a list of probably eligible patients (all scheduled cranial surgeries) is presented to the surgeon. After agreement by the surgeon, the patients are informed about the study.

### Assignment of interventions: allocation

#### Sequence generation {16a}—concealment mechanism {16b}—implementation {16c}

Patients are randomized into two groups, i.e., one arm treated with commercially available fibrin sealants and the other arm treated with L-PRF. The randomization sequence is generated by the study coordinator using an online randomization tool (www.sealedenvelope.com), for simple randomization with two treatment groups of equal size, no blocks, and no stratification factors. The allocation list is then uploaded into the Research Data Capturing solution (RedCap), a system where access is restricted to the study personnel. Only one subject’s allocation can be released at a time, after confirming that the patient is suitable for randomization. Participants are enrolled by the study coordinator or his/her delegate. Randomization is done during surgery using the Randomize-tool in RedCap by trained study personnel, other than operating room staff, for example, the study coordinator. The arm to which the patient is allocated is communicated by telephone to the operation theater staff.

### Assessment of interventions: blinding

#### Who will be blinded? {17}

The randomization is single-blinded, i.e., enrolled patients do not know which group they are allocated to. Blinding of surgical staff is not possible as L-PRF is clearly different from fibrin sealants. The bias of surgeons is reduced as much as possible by announcing the treatment arm only after the dural opening. Paramedics at the neurosurgery hospitalization department do not have access to the randomization code, thus reporting bias is reduced during hospital follow-up. Researcher’s bias is reduced by reblinding subject allocation after surgery. For analysis, group allocation will only be released after the statistical analysis has been performed.

#### Procedure for unblinding if needed {17b}

Unblinding may be done after completion of the outpatient follow-up visit (FU3) or in case of any serious adverse event that is probably related to the study.

### Data collection and management

#### Plans for assessment and collection of outcomes {18a}

Preoperative data is collected from the preoperative assessment of the anesthesiologist (medical history, comorbidity, and medication use) and neurosurgeon (surgical indication) available in the electronic patient files. Preoperative patient questionnaires are on paper source documents. Intraoperative data and used materials are collected from the neurosurgical report. Postoperative data during hospitalization is collected from hospitalization reports in the electronic patient files. Data from the outpatient follow-up are available in the electronic files. Postoperative questionnaires are filled out on paper source documents or electronically via RedCap. Data transfer from source documents to the RedCap data collection tool will be done by trained study personnel (research assistant). Regular data quality checks are performed in RedCap to ensure complete and accurate data transfer. Paper source documents will be stored for 10 years after completion of the study in a secure location at the study site.

#### Plans to promote participant retention and complete follow-up {18b}

The 6–12 weeks postoperative outpatient follow-up (FU3) is planned and notified to the patient before hospital discharge. Patients are requested to fill in the questionnaires at the 6–12 weeks postoperative visit (FU3). If the patient is not present at this appointment, or if the clinical follow-up appointment is rescheduled outside the FU3 visit time window (6–12 weeks postoperative), the patient is contacted by telephone to complete the questionnaires within the proposed time window. If this attempt is unsuccessful, the patient is considered lost to follow-up. In case of subject withdrawal, an exit note is added to the electronic case report form (e-CRF) mentioning the withdrawal date and reason of withdrawal.

#### Data management {19}

Data are collected partially on paper source documents (patient questionnaires) and electronic source documents (patient medical records containing surgical and hospitalization reports, registration of used materials). Data is collected by the principal investigator or his designee and locally entered into the e-CRF (RedCap). RedCap is primarily a data collection tool to facilitate post-study analysis based on qualitative data. Access to the e-CRF is strictly regulated and only possible with personal credentials obtained only after successfully completing a specific examination. All data operations are monitored and verified via a tracking system. Entries are verified by double entry (for example manual and automated summary of EQ-5D result); format checks, e.g., integer; and warning messages if data are outside an expected range of values.

As appropriate, baseline characteristics will be reported by mean and standard deviation or number and proportions. The effect of the intervention on the primary outcome will be assessed by comparing the proportion of patients presenting with a postoperative CSF leakage within 12 weeks after surgery.

#### Confidentiality {27}

Patients are identified by their unique study subject number, to ensure the subject’s pseudonymity. All data are processed without identifiable reference to the patient. One secured subject identification list is available in the investigator site file, stored at the study site, containing the code with the study subject number and patient’s name, birth date, and hospital number.

#### Plans for collection, laboratory evaluation, and storage of biological specimens for genetic or molecular analysis in this trial/future use {33}

Not applicable.

### Statistical methods

### Statistical methods for primary and secondary outcomes {20a}

Data will be analyzed in the GraphPad Prism software using a simple *t* test, or non-parametric Mann–Whitney *U* test when not normally distributed, with Bonferroni Holm correction for multiple testing. A *p*-value ˂ 0.05 is considered statistically significant. The primary outcome, i.e., CSF leakage, will be analyzed in terms of a difference in risk between the two treatment groups. The mean difference between the two treatment groups will be reported as a two-sided 95% confidence interval. Secondary endpoints will be calculated using a simple *t* test, non-parametric Mann–Whitney *U* test when not normally distributed, Fisher’s exact test, or chi-squared test, based on the number of events.

### Interim analyses {21b}

Per protocol, no interim analyses will be performed.

### Methods for additional analyses (e.g., subgroup analyses) {20b}

Subgroup analyses will be performed for surgical indication (tumor, vascular or functional), craniotomy diameter (small: ˂ 30 mm; medium: ˃ 30 mm; ˂ 80 mm; and large: ˃ 80 mm) recurrent surgery, patient age, gender, medication use, and alcohol and smoking habits. The data will be statistically tested using a simple *t* test or Mann–Whitney *U* test, or Fisher’s exact test or chi-squared test depending on normality, type of variable, and number of events. We do not intend to perform adjusted analyses.

### Methods in analysis to handle protocol non-adherence and any statistical methods to handle missing data {20c}

The results will be analyzed according to the “intention-to-treat” (all randomized participants, irrespective of protocol adherence), “per-protocol” (only participants that were treated according to the protocol), and “as-treated” (all participants according to the treatment they received) principles. These three analyses will be compared to show the possible impact of a lack of data on the results. For the primary endpoint, no missing data are expected. For secondary endpoints (cost-effectiveness evaluation), the predictive mean of the other values within the arm will be used.

### Plans to give access to the full protocol, participant-level data, and statistical code {31c}

The study protocol has been registered and is available at ClinicalTrials.gov (ID: NCT03812120). Statistical code and participant-level dataset will not be available.

### Oversight and monitoring

#### Composition of the coordinating center and trial steering committee {5d}

The trial protocol and CRF were designed by the principal investigator. Protocol revisions and changes to the CRF are managed by the principal investigator and study coordinator. The daily operation of the study is followed up by the study coordinator and regularly reported to the principal investigator and the sponsor. Annual progress and safety reports, including adverse events, are reported by the study coordinator. Trial progress and patient safety are assessed by a supervisory committee, consisting of specialists in the field of neurosurgery on a yearly basis. This meeting is organized by the study coordinator and principal investigator (trial management committee). The steering committee has approved the final version of the protocol, reviews the progress of the study, and, if necessary, agrees to changes to the protocol to ensure the efficient running of the study. The publication of study reports will be coordinated by the principal investigator and study coordinator.

#### Composition of the data monitoring committee, its role, and reporting structure {21a}

As no high-risk populations (e.g., children or pregnant women) are included in the study and as the study does not involve any important additional medical risk, no data safety monitoring board was deemed necessary.

#### Adverse events reporting and harms {22}

Harms and adverse events that may be expected in the study population include intracranial hemorrhage, tension pneumocephalus, wound problems, and neurologic complications such as seizures, dysphasia, or motor deficits [15]. During hospitalization, harms will be systematically collected from documentation of clinical and radiological examinations and daily nursing reports in the electronic patient files. At the outpatient follow-up (FU3), harms will be collected based on anamnesis and clinical examination. Additionally, four open-ended questions will be presented to the subjects:

- Did you suffer from any complaints?
- Have you been ill?
- Have you taken any medication?
- Were there any problems regarding wound healing?

During the study, all adverse events are recorded and notified to the sponsor with a yearly progress report. According to the local law, serious adverse events are reported immediately, after first knowledge, to the sponsor, accompanied by detailed written reports. A serious adverse event is defined as any untoward medical occurrence or effect that results in death, is life-threatening, requires hospitalization or prolongation of existing hospitalization, results in persistent or significant disability or incapacity, or is a congenital anomaly or birth defect. Serious adverse events defined as such will be reported in the final manuscript of the study. The sponsor shall keep detailed reports of all adverse events which are reported. For the reported death of a subject, the investigator shall supply the institutional Ethical Committee with any additional information requested.

#### Frequency and plans for auditing trial conduct {23}

No auditing visits are planned. However, routine audits can be announced by the sponsor to the principal investigator.

#### Plans for communicating important protocol amendments to relevant parties (e.g., trial participants, ethical committees) {25}

Protocol amendments shall be filed to the institution’s Ethical Committee for approval, after agreement of the principal investigator. If the amendment might affect the safety or procedure of already enrolled subjects, they will be notified and asked to sign an additional updated informed consent form.

### Dissemination plans {31a/b}

Upon finalization of the study, the institutional Ethical Committee will be notified. The results of the study will be submitted to a peer-reviewed scientific journal and presented at international conferences. The results will be disseminated regardless of the magnitude or direction of the effect. If requested by the patient, he/she will be updated about the results via e-mail when available.

Substantial contributions to the design, conduct, interpretation, and reporting of the clinical trial will be recognized through the granting of authorship on the final trial report. We do not intend to employ professional writers.

## Discussion

The design and rationale of a prospective randomized clinical trial studying the role of L-PRF in cranial dural closure are discussed. The primary aim of the study is to investigate the non-inferiority of L-PRF compared to commercially available fibrin sealants, the current standard of care. Additionally, we assess the safety and cost-effectiveness of L-PRF for dural closure in cranial surgery. The results of this trial will provide further insight into the efficiency of dura closure. As CSF leakage is an important complication of cranial surgery, this study is of major importance to the field of neurosurgery. Some preliminary pilot studies reported the feasibility of L-PRF as a closure aid in neurosurgery [9, 16]. L-PRF has been reported to enhance wound healing by providing growth factors to the surrounding tissue [17, 18]. This study represents the first large-scale randomized controlled trial comparing L-PRF to the current standard of care. With the intended sample size, we believe to be able to show the non-inferiority of L-PRF with respect to CSF leakage. The results of this trial can impact future decision-making for dura closure in cranial surgery.

## Trial status

Recruitment of this clinical trial started on January 6, 2020. We are currently recruiting under protocol version 8 (9 December 2021). Study completion is estimated by December 2023.

## Supplementary Information

**Additional file 1: Table S1.** World Health Organization Trial Registration Data Set {2b}.

**Additional file 2: Appendix 1.** Information sheet for the patient.

## Data Availability

The final dataset and study reports will be included in the Trial Master File for the study and archived at the sponsor and participating sites for 20 years after the study conclusion. The files will be accessible to dedicated personnel at the study site. The datasets used during and/or analyzed during the current study are available from the corresponding author upon reasonable request.

## Abbreviations

CSF: Cerebrospinal fluid
e-CRF: Electronic case report form
EMA: European Medicines Agency
EQ-5D: EuroQol Five Dimensions Questionnaire
FDA: Food and Drug Administration
FU: Follow-up
L-PRF: Leukocyte- and platelet-rich fibrin
RAND SF-36: Research and Development 36-Item Short Form Survey
REDCap: Research Data Capturing Solution
QALY: Quality-adjusted life-year
QoL: Quality of life
