# Supplementary material for: Leukocyte- and platelet-rich fibrin in cranial surgery: study protocol for a prospective, parallel-group, single-blinded randomized controlled non-inferiority trial {1}
Source: Trials. 2023 Mar 23;24:219. doi: 10.1186/s13063-023-07252-w (PMC10034240; doi:10.1186/s13063-023-07252-w)
Supplement: Supplementary file 2 — Additional file 2: Appendix 1. Information sheet for the patient. [file 13063_2023_7252_MOESM2_ESM.docx]

**Information sheet for the patient**

**Version 5, 9-12-2021**

Complete title of the study: L-PRF in cranial surgery: a prospective, randomized trial.

**1. Introduction**

We invite you to participate in a scientific study. Before that you decide, it is important that you understand why the research is being conducted and what it implies for you. Take the time to read the following information and discuss it with others if you desire to. The investigator or a member of his team will discuss the information in this document with you and is available to answer questions. Ask questions if anything is not clear or if you would like to have more information.

This information sheet gives you information about:

- The purpose of this research and what will happen to you if you participate

- More information about conducting the research

**2. What is the purpose of this study?**

Recent developments in various surgical techniques of the brain have led to the need for repair of tissue defects. These tissue defects are currently being repaired using foreign material and sealed with commercial tissue glue (Tachosil © and Tisseel ©)

Through this study we want to compare the efficacy and safety of a body-specific fibrin membrane containing leukocytes (white blood cells) and platelets (L-PRF) with those standard foreign materials. We also want to make an evaluation of the costs and the action of both techniques. Further, we are interested in the possible complaints that may arise after the operation.

This body-specific L-PRF is obtained after a simple processing of your blood (maximum 8 tubes) during the operation. The material is already frequently used in dental and orthodontic practice.

We aim to include 350 patients in this study, which will have a duration of four years.

**3. Why am I chosen?**

We invite you to participate in this study because you will undergo a brain surgery at the neurosurgery department shortly.

The brain and spinal cord are surrounded by a protective fluid, the cerebrospinal fluid. There is a hard membrane around this brain fluid. During the operation, the surgeon must ensure that the wound in the meninges is properly sealed so that no fluid can leak.

In order to do so, various techniques exist, commercial products (Tachosil © and Tisseel ©) as well as the body's own L-PRF. Both types are already used in our hospital. This study compares the efficacy, safety and cost analysis of L-PRF with the commercial products. We also want to register any complaints and complications that you may experience.

**4. Do I have to participate?**

You decide whether or not to participate. You hereby have time to think and ask questions. You can exit the investigation at any time. The decision to withdraw or the decision not to participate will not affect the standard care that you will receive.

**5. What will happen to me if I participate?**

If you decide to participate in this study, you will be asked to sign a consent form. Your participation starts from the moment you signed up and will end after your last follow-up visit (6 to 12 weeks after surgery).

It is currently not always known what the best way is to treat patients. Therefore, different treatments must be compared. In this case, the commercial standard products will be compared with the body's own fibrin membranes. Study participants are therefore randomized into 2 groups: a group that is treated with the body's own L-PRF and a group that is treated with the standard products. This is necessary to be able to compare both techniques. The classification is made entirely randomly and by chance. Your examining physician knows which treatment has been assigned to you, but you will not know this before the study is completed. Both techniques have already been used extensively with good results, at this hospital as well as internationally. However, a comparative study has never been conducted.

You can only participate in this study if during the closure of the wound, the examining physician finds a leak in the meninges that is suitable for potential treatment with L-PRF or standard commercial products. Your participation can therefore also be discontinued during surgery.

The study includes an appointment at the time of admission to the hospital (screening and baseline), two checks during your stay in the hospital (2 days after surgery and the day you are discharged from the hospital), and a check-up between 6 and 12 weeks after your surgery. This appointment is a standard check-up and would also be organized if you would not participate in the study. Consequently, there will be no extra investigations for this study.

What is expected on each visit?

-1. Screening visit: Demographic data and medical history are interviewed. Your quality of life is assessed using questionnaires.

0. Randomization (during surgery): In one half of the patients, the defect in the meninges, which is created at the start of the operation is closed with the body's own fibrin membranes. For the other half of the patients, this defect is closed with the traditional standard techniques, provided that foreign material / commercially available material is used (Tachosil© and Tisseel©). During the operation, blood will be collected (maximum 8 tubes) for the production of the fibrin membranes. If you are assigned to the treatment with the commercial material, we may further investigate the autologous fibrin materials in the laboratory to gain a better understanding of the exact mechanism of action of these materials.

A member of the research team gathers information about the extent to which the brain fluid leak is sealed, general details about the operation, and any side effects or complications that may occur.

1. After surgery: You will stay in the hospital to recover for as long as necessary for patients undergoing this type of operation. Two days after the operation and shortly before discharge from the hospital, the wound is examined. Any side effects or complications are also recorded.

2. Follow-up visit: Between 6 and 12 weeks after the operation you will be invited to come to the hospital for a physical examination of the surgical wound, a check of any medicines that you have taken and any complaints that have occurred since the operation. Your quality of life is assessed by means of questionnaires.

The collected biological samples will be managed by the UZ/KU Leuven Biobank and will be processed immediately in the operating theatre or in the Allergy and Clinical Immunology lab of KU Leuven. These samples will be used or analyzed in view of the objectives of the study. A coding procedure is also used for the samples, just as for your medical data. The samples handed over to the sponsor will therefore only have an identification code within the framework of this clinical study. The medical examiner is responsible for ensuring the traceability of your biological samples. The biological material is considered a "gift" and you should be aware that in principle you will not receive any financial benefit (royalties). If you withdraw your consent, you have the right to have the collected biological samples destroyed. However, samples that were already used in the study at the time of withdrawal of consent cannot be retroactively destroyed. Data obtained from the analysis of the samples in question also remain the property of the client, in order to guarantee the validity of the study.

**6. Insurance**

An insurance policy has been taken out in the event that you sustain damage as a result of your participation in this clinical study. In accordance with the Belgian law of 7 May 2004 on experiments on the human person, the client is, even without error, liable for all damage incurred by the participant and which is directly or indirectly related to the experiment. The principal of this study (UZ Leuven) has taken out insurance that covers this liability. If you sustain damage as a result of your participation in this study, this damage will therefore be reimbursed to you or to your beneficiary in accordance with the Belgian law of 7 May 2004.

Contact details Insurer: Amlin Corporate Insurance, policy no. 299.053.700, contact details estate agent: Vanbreda Risk & Benefits, Plantin and Moretuslei 297, 2140 Antwerp.

**7. Costs and reimbursement**

All examinations and contacts are part of the standard care program. You will therefore not receive a reimbursement for your participation to this study.

**8. What are possible disadvantages of participation?**

You are undergoing a planned surgery. Possible risks and inconveniences that can be expected consist of the normal risks and inconveniences of the surgery that you will undergo. No side effects are expected. The L-PRF that will be used is specific to the body. This means that it is made from your blood and that there is hardly any chance of rejection.

**9. What are possible advantages of participation?**

You may not have a direct benefit from participating in this study.

The information that we will obtain from this study can help us to optimize treatment for future patients who need the type of surgery that you will receive. For example, we expect a possible faster recovery with one of the two techniques.

**10. Will my participation to this study be kept confidential?**

Yes. We follow ethical and legal guidelines and all information about your participation in this research is kept confidential. If you agree to participate in this study, you will receive a unique study number, which is then used for your identification in the research database. Your identity remains confidential. With your permission to participate in this study, you agree to the inspection of your medical record by your investigating doctor or study nurse and possibly the relevant governmental health authorities. The purpose of this medical file check is to ensure that your information is correctly recorded in the database. All these parties have a duty of confidentiality towards you as a research participant and nothing from which your identity can be revealed is disclosed outside the research center.

Your data is stored in accordance with EU Regulation 2016/679 or GDPR (General Data Protection Regulation). With permission to participate in this research, you agree that your coded data will be stored in accordance with this law.

If you have any further questions or comments, you can always contact the local Data Protection Officer (for UZ Leuven this is: [gdpr.research@uzleuven.be](mailto:gdpr.research@uzleuven.be)).

You have the right to make a complaint about how your information is treated with the Belgian supervisory authority responsible for enforcing data protection legislation: Data protection authority (GBA), Drukpersstraat 35 1000 Brussels, tel. +32 2 274 48 00, contact@apd-gba.be, [www.gegevensbeschermingsautoriteit.be](http://www.gegevensbeschermingsautoriteit.be)

**11. What happens with the results of the scientific research?**

The results can be published but your identity is not disclosed. If the results are published, you can obtain a copy of the results on request from the research doctor.

**12. Who checked the investigation?**

Every research in Belgium is assessed by an independent group of people in a so-called ethics committee for the protection of your safety, rights, well-being and dignity. This research has been evaluated and positively assessed by the Ethics Committee Research UZ / KU Leuven.

**13. Contact information**

If you wish to contact us if you have problems or concerns during the study:

Responsible for the study: Drs. Birgit Coucke, telephone 016/34 55 70

Principal investigator: Prof. dr. Dr. T Theys, telephone 016/34 42 90 (Neurosurgery Secretariat)

If you have any questions regarding your rights as a participant in the study, you can contact the ombudsman service in your hospital on telephone number 016/34 48 18. If necessary, the ombuds service can put you in contact with the Ethics Committee UZ / KU Leuven.

**Thank you for taking the time to read this information brochure.**

**Consent form (for patient)**

**The role of L-PRF in cranial surgery**

- I confirm that I have read and understood the information sheet version 5, 9-12-2021, for the study mentioned above. I have had the opportunity to consider the information and ask questions, and these questions have been answered to my satisfaction.
- I understand that my participation is voluntary and that I can stop at any time, without giving a reason and without affecting my medical care or legal rights.
- I understand that relevant parts of my medical file can be viewed by the research team of the neurosurgery department UZ Leuven, or from supervisory authorities where relevant for participation in this study.

I give permission to those people to view my file.

- I have received a copy of the information form for the participant, as well as this consent form.
- I agree to participate in the study mentioned above.
- **I agree to participate in this register and have given this permission before any study procedures are carried out.**

Patient:

______________________ ___________________ __________________

Name Signature Date

Legal representative if the patient is unable to give permission:

______________________ ___________________ __________________

Name Signature Date

Investigator or delegate:

I discussed the patient's information and consent form with the above-mentioned person before study procedures took place. I asked if he / she had any questions, and I answered the questions asked.

______________________ ___________________ __________________

Name Signature Date

**Consent form (for investigator)**

**The role of L-PRF in cranial surgery**

- I confirm that I have read and understood the information sheet version 5, 9-12-2021, for the study mentioned above. I have had the opportunity to consider the information and ask questions, and these questions have been answered to my satisfaction.
- I understand that my participation is voluntary and that I can stop at any time, without giving a reason and without affecting my medical care or legal rights.
- I understand that relevant parts of my medical file can be viewed by the research team of the neurosurgery department UZ Leuven, or from supervisory authorities where relevant for participation in this study.

I give permission to those people to view my file.

- I have received a copy of the information form for the participant, as well as this consent form.
- I agree to participate in the study mentioned above.
- **I agree to participate in this register and have given this permission before any study procedures are carried out.**

Patient:

______________________ ___________________ __________________

Name Signature Date

Legal representative if the patient is unable to give permission:

______________________ ___________________ __________________

Name Signature Date

Investigator or delegate:

I discussed the patient's information and consent form with the above-mentioned person before study procedures took place. I asked if he / she had any questions, and I answered the questions asked.

______________________ ___________________ __________________

Name Signature Date
